# Differences in Knowledge, Stress, Sensation Seeking, and Locus of Control Linked to Dietary Adherence in Hemodialysis Patients

**DOI:** 10.3389/fpsyg.2016.01864

**Published:** 2016-11-29

**Authors:** E. Leigh Gibson, Ines Held, Dina Khawnekar, Peter Rutherford

**Affiliations:** ^1^Department of Psychology, Whitelands College, University of RoehamptonLondon, UK; ^2^Renal Unit, Wrexham Maelor HospitalWrexham, UK; ^3^North East Essex Diabetes ServicesColchester, UK; ^4^Quintiles, ReadingUK

**Keywords:** hemodialysis, adherence, diet, stress, locus of control, personality

## Abstract

Patients with chronic kidney disease (CKD) often require regular hemodialysis (HD) to prolong life. However, between HD sessions, patients have to restrict their diets carefully to avoid excess accumulation of potassium, phosphate, sodium, and fluid, which their diseased kidneys can no longer regulate. Failure to adhere to their renal dietary regimes can be fatal; nevertheless, non-adherence is common, and yet little is known about the psychological variables that might predict this dietary behavior. Thus, this study aimed to assess whether dietary adherence might be affected by a variety of psychological factors including stress, personality, and health locus of control, as well as dietary knowledge, in chronic HD patients. Fifty-one patients (30 men; age range 25–85) who had undergone HD for at least 3 months and had been asked to restrict at least one of potassium, phosphate or fluid, were recruited from a hospital renal unit. Measures of adherence to each of potassium, phosphate, and fluid were derived from standard criteria for these physiological indices in renal patients. Knowledge of food/drink sources of these dietary factors, and their medical implications in relation to HD and CKD were assessed by a bespoke questionnaire. Psychological factors including stress, personality and health locus of control beliefs were measured by standardized questionnaires. Having to restrict a particular nutrient was associated with better knowledge of both food sources and medical complications for that nutrient; however, greater dietary knowledge was not linked to adherence, and knowledge of medical complications tended to be associated with poorer adherence to potassium and phosphate levels. Adherence to these two nutrient requirements was also associated with lower reported stress in the past week. Adherence was associated with differences in locus of control: these differences varied across indices although there was a tendency to believe in external loci. For potassium, phosphate, and fluid restriction, adherers were less likely to be sensation seekers but did not differ from non-adherers on impulsivity, anxiety sensitivity, or hopelessness. In conclusion, the links between dietary adherence and stress, locus of control and personality suggests that screening for such psychological factors may assist in managing adherence in HD patients.

## Introduction

Chronic kidney disease (CKD) is a common long-term condition which as it progresses requires intensive nursing, medical, and dietary interventions, with considerable variability in patient outcomes (Junaid Nazar et al., 2014). As kidney function declines, renal replacement therapy is required: in Europe, 340,000 patients are receiving regular hemodialysis (HD) or peritoneal dialysis (PD) (European Renal Care Providers Association, 2013).

For many patients with advanced kidney failure, foods that are (or are perceived to be) healthy for the majority of people turn into potential “deadly sins.” As kidney function deteriorates, the ability to excrete potassium (K), phosphate (PO_4_), and sodium (Na) from the blood is reduced and fluid balance is difficult to maintain as excess fluid cannot be lost. This means that patients have to limit their intake of foods and fluids in order to maintain safe levels (Palmer et al., 2015).

For instance, raised blood K levels (hyperkalemia) from fruits, vegetables, and nuts can lead to sudden cardiac arrest by causing arrhythmias. PO_4_, for example, from meat and dairy products, promotes vascular calcification and increases cardiovascular risk (Beto and Bansal, 2004). Vascular calcification also reduces the surgical success rate for kidney transplantation (Tentori et al., 2008).

Dialysis only partially replaces kidney function in terms of solute removal and fluid balance, and with hospital based HD, this is done typically three times a week. For HD patients, fluid balance becomes a significant challenge as their urine output tends to deteriorate quickly: the interdialytic weight gain (IDWG) can be significant, and higher levels are associated with increased blood pressure and increased mortality (Beto and Bansal, 2004). The effects of reduced Na excretion interact with those of water, increasing the risk of cardiovascular disease.

Renal dietitians work with the patient to keep K, PO_4_, and Na intake safe and to maintain fluid balance, but also to encourage an adequate intake of energy, macro- and micronutrients. However, there are several objective obstacles to adherence to intake recommendations:

1. Information regarding K and PO_4_ content is not readily available on food packaging.
2. Recommendations aimed at limiting water, PO_4_, and K intake can contradict those for pre-existing conditions or general health received in the past from healthcare professionals and media.
3. Obesity is a risk factor for CKD (Hsu et al., 2006), and obese patients are advised to lose weight in order to be fit for a kidney transplant.
4. Dietary changes need to be individualized for each patient and will vary over time, and this complex information has to be processed and utilized on a background of increased stress levels with the effects of kidney failure and its treatment.
5. CKD and specifically HD can adversely affect cerebral function and cause cognitive defects long-term and acutely during the treatment (McIntyre, 2010).

There is evidence that HD patients are often not successful in achieving dietary adherence: for example, in the most recent UK Renal Registry report, 30% had levels above the target PO_4_ range. On the other hand, malnutrition is also observed commonly in HD patients and is also associated with increased mortality (Pifer et al., 2002) and is multifactorial in origin (Chazot, 2009). Moreover, although the data are limited, there is evidence of links between psychosocial factors (including depression) and malnutrition, serum K, PO_4_, and IDWG (Sensky et al., 1996; Khalil et al., 2011).

Despite this evidence linking eating behaviors and important clinical outcomes in HD patients, the impact of standard clinical interventions in improving nutrition and dietary control remains disappointing. In the DOPPS study, significant numbers of patients exhibited non-adherence to standard measures of dietary control including high IDWG (11%), high PO_4_ levels (12.8%), and high pre-dialysis K levels (20%) (Tentori et al., 2008). Importantly, Durose et al. (2004) examined relations between dietary adherence and knowledge of the required dietary restrictions and the medical complications of dietary non-adherence in CKD: not only was patient knowledge not predictive of improved dietary adherence, but conversely greater knowledge of dietary PO_4_ sources, and of the medical complications of non-adherence was associated with poorer adherence, at least for PO_4_ and Na or fluid restrictions.

Additionally, despite a wealth of evidence for the importance of psychosocial variables in health behavior (Michie et al., 2011), and recognition of the need for psychological interventions to support long-term behavior change in CKD patients (Clark et al., 2014), a recent systematic review of adherence in PD patients revealed a scarcity of evidence for the role of psychosocial factors in dietary adherence in these patients (Griva et al., 2014).

Psychological distress has been proposed as a risk factor for poor outcomes in CKD patients (Zalai et al., 2012) with even minor stressors having significant impact on an individual’s well-being and health outcomes (Hamer et al., 2006). For example, the frequency of stressful events assessed by the Weekly Stress Inventory (WSI) was associated with medical regimen adherence over 4 weeks in HD patients indicated by blood K and urea nitrogen levels (Hitchcock et al., 1992), and stress measured by the similar Daily Stress Inventory was associated with poor fluid intake adherence (higher IDWG) in patients with end stage renal disease (ESRD) (Everett et al., 1995). Stress makes dietary change difficult, as food choices tend to revert to palatable, rewarding sweet and fatty food, and moreover the impact of stress on health behaviors may be mediated by personality traits that promote risky behaviors as coping mechanisms (Gibson, 2012). Furthermore, food and drug habits share underlying biological and psychological processes (Volkow et al., 2011), so that factors that affect drug addiction may also be relevant for dietary behavior. For example, high levels of smoking were associated with failure to adhere to dietary and fluid restrictions in an HD population (Kugler et al., 2005).

Recent models of behavior change are relevant to dietary adherence (Atkins and Michie, 2015), and suggest that predictors of successful change will include variables that influence one’s sense of identity, belief in one’s capabilities, the importance of achieving the relevant dietary goals, but also more fundamental traits that affect responses to urges and momentary decisions, such as inhibitory control, impulsivity, and reward sensitivity (Michie et al., 2011). Taking such factors into account may allow improvement of psychological interventions with broader promotion of adherence than previously seen (Sharp et al., 2005), particularly as studies of psychosocial factors have usually focused on fluid adherence (Friend et al., 1997; Howren et al., 2016b).

The present study was conducted to identify whether dietary knowledge and knowledge of medical complications influenced adherence measures in HD patients, using an improved assessment of such knowledge. In addition, this study sought to identify whether key psychological variables such as perceived stress, health locus of control, and personality variables associated with lack of control over eating or other habitual behaviors predicted dietary adherence as measured by K, PO_4_ and fluid indices.

## Materials and Methods

### Design

The study was a cross-sectional single-center survey involving single-point questionnaire data collection measuring aspects of knowledge of complications of renal disease and concomitant dietary advice as well as facets of personality and psychosocial attitudes, for comparison with routine dialysis outcomes used to indicate the degree of dietary adherence, in a group of HD patients.

### Participants

Patients on HD for at least 3 months were identified and recruited for the study from the Renal Unit at North East Wales NHS Trust, Wrexham Maelor Hospital. This was considered to be a sufficient period in principle to allow patients to adapt their eating habits to their new requirements, following dietetic advice. Patients who were hospitalized within the last 3 months and who were on dialysis for less than 3 months or with any concomitant illness were excluded from the study.

The recruitment target was 50 patients, based on power calculations from results of Durose et al. (2004), since an adapted version of their survey of knowledge of dietary restrictions for dialysis has been used. For significance level of alpha = 0.05, sample sizes of between 20 and 60 participants, using G^∗^Power (Erdfelder et al., 1996) were required, determined from several chi-square results relating knowledge to adherence. Similarly, Hitchcock et al. (1992) found that stress was significantly correlated to K levels (partial *r* = 0.394), in a study of 55 HD patients, while Everett et al. (1995) demonstrated that stress was significantly associated with higher IDWG in a sample of 42 patients.

Ethical approval was received from the North East Wales Local Research Ethics Committee and University of Roehampton, and all participants gave signed informed consent in accordance with the Declaration of Helsinki. Following consent, patients were asked to complete five questionnaires whilst at home on a non-dialysis day and to return them on the following dialysis session.

### Questionnaires

#### Modified Renal Knowledge Questionnaire

The following variables were determined using the Renal Knowledge Questionnaire (adapted from Durose et al., 2004, with the authors’ permission) for participants on HD: (i) demographic characteristics such as age, gender, marital status, ethnicity, employment status and occupation, dialysis period, special dietary preferences, dietary advice received from renal dietitian, and prevalence of morbidity such as diabetes mellitus; (ii) variables pertaining to the knowledge of restricted foods and nutrients, reasoning behind the restrictions (i.e., medical consequences of not adhering) and sources of foods high in K, PO_4_, fluid, and sodium; (iii) three additional questions were designed to measure the participants’ attitudes toward the dietary advice and having to restrict their diets. Participants rated attitudinal statements from “definitely true” to “definitely false” on a 4-point Likert scale.

A number of other modifications were made to phrasing of other items and there was an important adjustment to the knowledge variables scoring method: Durose et al. (2004) attributed scores as: correct response = 1; incorrect response = 0; unsure = 0, but the present study used a method of negative scoring for incorrect answers (Gibson et al., 1998). This method prevented artificially high scores due to blanket responding, such that random responding would tend to sum to 0.

##### Scoring knowledge of food sources of nutrients

Each question asking “Which of these foods are high in…?” had seven foods to choose from: for K, PO_4_, and fluid, five foods were high sources; for salt, four foods were high sources. Correct answers were scored with +1, whereas either incorrect answers or “don’t know” were scored with -1: thus, the possible summed scores ranged from -7 to +7 for each question, in steps of 2, as each correct answer replaced a negatively scored incorrect one.

In addition, for comparison of knowledge across nutrients, participants were classified into those with “good” or “poor” knowledge, where good knowledge represented correct responses in at least five out of seven foods (all participants answered these questions). Although Durose et al. (2004) divided good and poor knowledge at the mid-point of their scale, and found that PO_4_ food knowledge was worse in PO_4_ adherers, we chose five out of seven as providing a slightly higher threshold for “good” knowledge, to reduce the chance that the adverse knowledge-adherence relationship could be artefactual.

##### Scoring of knowledge of medical complications of nutrients

Each question asking “Do you know why … is relevant to your diet?” and one asking “Do you know why you need to reduce your fluid intake?” had six responses to choose from, in terms of impacts on health, and “Don’t know.” For K, only one answer was true, for PO_4_ and Na, two were true, and for fluid, three were true. Correct answers (i.e., ticking the true answer and not ticking the false answers) were scored as +1, whereas incorrect answers were scored with -1. Endorsing “don’t know” was given a score of -6, to be equivalent to getting all six answers wrong: thus, the possible summed scores ranged from -6 to +6 for each question, in steps of 2, as each correct answer replaced a negatively scored incorrect one.

#### Weekly Stress Inventory-Short Form

The WSI-Short Form (WSI-SF) is a 25-item self-report scale that measures the number of minor stressors that occur in 1 week (Brantley et al., 2007), and that has been associated with protein and potassium indicators of dietary adherence over 4 weeks (Hitchcock et al., 1992). The WSI-SF assesses minor stressors over the previous week, including the number of stressful events and perceived impact of those events, i.e., individuals rank items on an 8-point Likert scale, with values ranging from 0 (did not occur) to 7 (extremely stressful). Brantley et al. (2007) proposed two scores, the event score (WSI-E) which is the total number of events occurring, and the impact score (WSI-I), which is the sum of ratings of distress on the event scores. However, WSI-I is therefore a function of WSI-E, such that typically the more events there are, the higher the sum of distress ratings is likely to be – in our sample these measures correlate strongly (Pearson’s *r* = 0.90, *p* < 0.001); on the other hand, this WSI-I score would not discriminate between a participant experiencing a minimally stressful event every day from one who experienced a single extremely stressful event in that week. Therefore, we opted to use the average of the ratings of distress for those events that occurred, as better capturing the “impact” of stress that week; we termed this variable “WSI-mean.”

#### Perceived Stress Scale Questionnaire

The Perceived Stress Scale Questionnaire (PSS) is a brief questionnaire designed to measure the degree to which situations in one’s life are appraised as stressful (Cohen et al., 1983); the scale has been used previously in HD patients (Garcia-Llana et al., 2013). The 10-item version has been used, as it has the best psychometric properties. The questions ask about how an individual felt in the previous month. In each case, patients were asked to indicate how often they felt or thought in a particular way. The frequency scale range was 0 = never, 1 = almost never, 2 = sometimes, 3 = fairly often, 4 = very often. PSS-10 scores were obtained by reversing the scores on the four positive items (items 4, 5, 7, and 8), and then summing across all 10 items. A higher score was indicative of a greater degree of perceived stress.

#### Multidimensional Health Locus of Control-C

The Multidimensional Health Locus of Control 18-item Form C is specifically designed for use in patient populations suffering from chronic medical conditions (Wallston et al., 1994), and has recently been linked to fluid adherence in HD patients (Howren et al., 2016a). The scales measure four types of beliefs about health locus of control, i.e., where in their internal and external psychosocial world the patient believes control over their health outcome lies: Internal control (perceived self-control; items 1, 6, 8, 12, 13, 17; e.g., “I am directly responsible for my condition getting better or worse.”); Chance (2, 4, 9, 11, 15, 16; e.g., “Most things that affect my condition happen to me by chance.”); Doctors (3, 5, 14; e.g., “Following doctor’s orders to the letter is the best way to keep my condition from getting any worse.”); Other people (7, 10, 18; e.g., “The type of help I receive from other people determines how soon my condition improves.”). The scores were created by summing, for each appropriate set of items, the responses given on a 6-point scale, from 1 = strongly disagree to 6 = strongly agree. No items were reversed before summing.

#### Substance Use Risk Profile Scale

Substance Use Risk Profile Scale (SURPS) is a revised, shortened 23-item version of Drug Abuse Subtyping Scale used as a measure for determining personality variables related to substance abuse risk or health behavior problems (Woicik et al., 2009). Each item was rated on the scale: strongly disagree = 1, disagree = 2, agree = 3, strongly agree = 4, such that higher scale scores represent a greater level of that trait. Four subscales measured hopelessness (items 1, 4, 7, 13, 17, 20, 23; range 7–28), anxiety sensitivity (items 1, 10, 14, 20, 21; range 5–20), impulsivity (items 2, 5, 11, 15, 22; range 5–20), and sensation seeking (items 3, 6, 9, 12, 16, 19; range 6–24). Items 1, 4, 7, 13, 20, 23 were reversed. We hypothesized that adherers would score lower on all of these personality risk factors for substance abuse. This scale was chosen for its brevity and association of some of the traits measured with adherence (Garcia-Llana et al., 2013), although the SURPS itself had not previously been used in HD patients.

### Adherence Assessment

Biochemical measures of serum K and PO_4_ have been widely used as indicators of dietary adherence (Christensen et al., 1992; Kugler et al., 2005). In the present study, routine predialysis blood results for three consecutive months were used for analysis to determine adherence behavior. Dietary adherence was defined as K < 6.0 mmol/L, PO_4_ < 1.8 mmol/L and or weight gain (IDWG) < 2.0 kg in three “short” dialysis intervals. For example, values of biochemical measures were taken one for each month (June/July/August), and analyzed individually for each session to determine adherence status on each occasion. Height and weight measurements were computed with the help of calibrated electronic scales and a stadiometer; body mass index (BMI) was calculated as weight (kg) divided by height (m^2^). IDWG was obtained by computing the difference between patient weight immediately prior to a dialysis session and patient weight following completion of the previous dialysis session. Thus, IDWG provides an indicator of adherence to fluid restrictions (Kugler et al., 2005). The final “adherer” (scored as 1) vs. “non-adherer” (scored as 0) classification was defined as being within the adherence criteria for all three measurement points, within each variable, i.e., K, PO_4_, and IDWG (fluid). This dichotomized cut-off approach was similar to that taken by Durose et al. (2004) and has practical validity for HD patient management since there is a regular monitoring of clinical standards of these same parameters in a dichotomous fashion for comparative clinical audit and hospital performance management purposes (Pifer et al., 2002).

### Data Analysis

All analyses were carried out using SPSS software (v19-21). Categorical data were analyzed by chi-square. Scale score group means were compared by non-parametric Mann–Whitney *U* tests, due to some non-normality of score distributions. Significance levels (alpha = 0.05) were considered as two-tailed, unless otherwise stated.

## Results

### Patient Characteristics

Fifty-five patients were recruited and completed all measures but analyses were conducted on the 51 patients who were being asked to restrict at least one of either K, PO_4_, or fluid. Fifty-nine percent were male (*n* = 30) and 41% female (*n* = 21), with age range 25–85 years: the majority (53%; *n* = 27) were 65 or over, with 18% (*n* = 9) being 55–64 and 29% (*n* = 15) being 25–54 years old. All but one of the participants was white, only 10% described themselves as single, and 96% had children: 53% owned their own home. The majority (61%) of the participants were retired, while 18% were medically unfit for work.

Most of the participants had a normal or overweight BMI (kg/m^2^; 75%: overall mean ± SD = 27.7 ± 7.3); however, 25% of the sample were clinically obese. Fourteen patients (28%) were had diabetes. All patients had been receiving HD for more than 3 months (mean 48.1 ± 42.5 months) and HD duration did not differ between adherers and non-adherers for PO_4_ or fluid restrictions: however, for those restricting K, adherers had been on dialysis for less time than non-adherers (43.9 ± 50.6 vs. 64.7 ± 38.2 months; *U* = 80, *p* < 0.05).

### Knowledge of Renal Diet

#### Food Sources

The proportions of the participants demonstrating good knowledge (five out of seven correct) vs. poor knowledge of sources of K, PO_4_, Na, and fluid in foods are shown in **Table 1**. As would be expected given the sensory cues, patients were better at knowing foods that were high in Na or fluid; however, the distributions were only significantly different for knowledge of fluid sources.

**Table 1:** Distribution of “good” and “poor” knowledge of food sources high in potassium, phosphate, sodium, and fluid (*n* = 51).

	Food sources high in:			
Knowledge level^a^	Potassium	Phosphate	Sodium	Fluid
Good (%)	45	40	62	82
Poor (%)	55	60	38	18
*p*-value (χ^2^ test, two-tail)	0.12	0.07	0.07	0.001

*^a^Good knowledge represented correct responses for at least five out of seven foods; all participants completed these questions.*

K, PO_4_, and fluid food source knowledge levels were tested for any difference between those required to limit intake of the nutrient and those who were not (**Table 2**: Mann–Whitney *U* tests). Those restricted on K and fluid had better knowledge of foods high in the respective nutrients than those not restricted (*U* = 143.5, *p* < 0.05; *U* = 125, *p* < 0.025, respectively). Those restricted on PO_4_ did not have significantly better knowledge of food sources (*U* = 94.5, *p* > 0.05), although the number of patients restricting PO_4_ was low. Finally, patients who were required to restrict K, PO_4_, and fluid tended to have better summed knowledge of which foods were high in those nutrients [*n*, mean ± SD: 28, 6.1 ± 8.6 vs. 23, 1.5 ± 9.4] but the difference did not reach significance (*U* = 233.5, *p* < 0.10, two-tail).

**Table 2:** Differences in knowledge of food sources high in a nutrient for participants restricted or not restricted in that nutrient or who are or are not adherent (mean ± SD).

		Knowledge score (-7 to +7) for food sources high in:		
Group		Potassium	Phosphate	Fluid
Nutrient restricted?				
Yes		1.4 ± 4.2	0.7 ± 3.4	4.2 ± 3.4
	n	38	45	39
No		-1.6 ± 4.1^∗^	-1.7 ± 4.8	-1.0 ± 5.8^∗^
	n	13	6	12
Dietary adherence?				
Yes		1.1 ± 4.3	0.5 ± 3.6	3.1 ± 4.7
	n	23	28	14
No		1.9 ± 4.1	1.0 ± 3.2	4.8 ± 2.2
	n	15	17	25

*^∗^*p* < 0.05, two-tail; more positive scores represent greater knowledge.*

*“Nutrient restricted” refers to the fact that the patient was being asked to restrict at least one of K, PO_*4*_, or fluid. Dietary adherence was defined by cut-offs relevant to each nutrient (see Materials and Methods; Adherence Assessment).*

Examining only those patients who had to restrict either K, PO_4_, or fluid, knowledge scores were compared between those who did and did not adhere to the dietary restriction. There were no differences between these adherence groups in knowledge of the restricted nutrient (**Table 2**).

#### Medical Complications

Patients required to restrict particular nutrients had better knowledge of the particular medical complications that are associated with failure to adhere to those nutrients compared to those not needing to restrict the nutrients (**Table 3**).

**Table 3:** Differences in knowledge of medical complications of nutrients for participants restricted or not restricted in that nutrient or who are or are not adherent (mean ± SD).

		Knowledge score of medical complications of each nutrient (-6 to +6):		
Group		Potassium	Phosphate	Fluid
Nutrient restricted?				
Yes		2.2 ± 4.6^∗^	0.1 ± 4.4^∗∗^	1.2 ± 3.0^∗^
	n	38	45	39
No		-0.5 ± 4.6	-5.3 ± 1.6	-2.6 ± 4.4
	n	13	6	7
Dietary adherence?				
Yes		1.5 ± 4.4	-1.3 ± 4.3^∗∗^	1.3 ± 3.3
	n	23	28	14
No		3.3 ± 4.9	2.4 ± 3.7	1.2 ± 2.8
	n	15	17	25

*^∗^*p* < 0.05, ^∗∗^*p* < 0.01 two-tail; more positive scores represent greater knowledge.*

*“Nutrient restricted” refers to the fact that the patient was being asked to restrict at least one of K, PO_*4*_, or fluid. Dietary adherence was defined by cut-offs relevant to each nutrient (see Materials and Methods; Adherence Assessment).*

By contrast when comparing adherers and non-adherers in knowledge of medical consequences, K adherers tended to have worse knowledge than non-adherers (**Table 3**), and for PO_4_, knowledge of medical complications was significantly worse for adherers.

#### Psychological Factors Associated with Dietary Adherence

Each of the psychological variables was tested for a link to adherence by examining whether adherers on each nutrient differed significantly from non-adherers on each variable score (Mann–Whitney *U* test).

#### Stress

Stress was assessed by using the WSI-mean (from WSI-SF), which represents the average distress rating for the stressful events experienced over the previous week. For K (mean ± SD: 1.3 ± 1.3 vs. 2.5 ± 1.6, *p* < 0.05) and PO_4_ (1.3 ± 1.2 vs. 2.6 ± 1.5, *p* < 0.05), adherers reported significantly less stress than non-adherers. Stress was lower but not significantly different between fluid adherers and non-adherers (1.3 ± 1.8 vs. 1.9 ± 1.4). These differences were seen despite relatively low average distress ratings, i.e., even average stress for the non-adherers was below the scale midpoint. This low level of stress may in part explain the lack of any significant differences within the PSS-10 stress score, which requires a greater retrospective recall of stress, over 1 month, and so may be more susceptible to inaccurate recall.

#### Health Locus of Control and Attitude to Dietary Adherence

For K, adherers reported significantly lower levels of “Internal” locus of control (perceived self-control), but higher levels of locus of control in “Other people” (**Table 4**): locus of control in “Doctors” did not differ between potassium adherers and non-adherers. In contrast, PO_4_ adherers did not differ in “Internal” locus of control, but reported significantly higher levels of locus of control in “Doctors” and “Other people” (**Table 4**). Fluid adherers reported higher locus of control in “Doctors,” but did not differ for that in either “Other people” or “Internal” control (**Table 4**).

**Table 4:** Differences in health locus of control for participants who did or did not adhere to K, PO_4_, and fluid restriction (mean ± SD).

		Health Locus of Control^a^		
	*n*	Internal (range 6–36)	Doctors (range 3–18)	Other people (range 3–18)
K adherence (<6.0 mmol/L)				
Yes	22	18.8 ± 5.3^∗^	16.1 ± 2.5	15.1 ± 2.8^∗∗^
No	15	23.2 ± 5.7	14.7 ± 2.9	11.8 ± 4.2
PO_4_ adherence (<1.8 mmol/L)				
Yes	27	20.3 ± 4.5	16.3 ± 2.3^∗∗^	14.5 ± 3.3^∗^
No	17	22.6 ± 6.1	14.2 ± 2.9	12.3 ± 3.6
Fluid adherence (IDWG < 2 kg)				
Yes	25	21.4 ± 5.3	16.9 ± 1.6^∗^	13.6 ± 3.6
No	14	22.0 ± 5.9	14.9 ± 2.8	15.1 ± 2.4

*^a^Excluding Chance, which never approached significance; higher scores represent greater belief in that locus of control. For details of adherence criteria, refer to Section “Adherence Assessment.” ^∗^*p* < 0.05, ^∗∗^*p* < 0.01, two-tail.*

#### Personality Traits and Dietary Adherence

Four traits derived from the SURPS questionnaire, and considered as potential risk factors for non-adherence (sensation seeking, impulsivity, anxiety sensitivity, and hopelessness), were compared for differences between adherers and non-adherers (Mann–Whitney *U* tests). For K, PO_4_, and fluid restriction, adherers were significantly lower on sensation seeking but did not differ from non-adherers on impulsivity, anxiety sensitivity, or hopelessness (**Table 5**).

**Table 5:** Comparison of personality traits between adherers and non-adherers for potassium, phosphate, and fluid restrictions (mean ± SD).

Restriction	B	*n*	Sensation seeking (range 6–24)	Impulsivity (range 5–20)	Anxiety sensitivity (range 5–20)	Hopelessness (range 7–28)
Potassium	Yes	23	11.0^∗^ ± 3.5	10.1 ± 3.2	13.8 ± 2.8	15.0 ± 3.2
	No	15	13.5 ± 3.8	11.3 ± 2.8	12.9 ± 1.8	15.3 ± 4.1
Phosphate	Yes	28	10.8^∗∗^ ± 3.1	10.7 ± 2.9	13.8 ± 2.6	14.0 ± 3.1
	No	17	13.3 ± 3.4	11.8 ± 2.5	13.1 ± 2.0	16.0 ± 3.4
Fluid	Yes	14	9.1^∗∗^ ± 2.9	9.4 ± 2.3	13.3 ± 3.0	13.9 ± 3.2
	No	25	12.2 ± 3.9	11.4 ± 3.2	13.6 ± 2.1	14.4 ± 3.4

*^∗^*p* < 0.05, ^∗∗^*p* < 0.01 (one-tail, adherers vs. non-adherers). For all personality variables, higher scores indicate greater levels of those traits.*

## Discussion

This study examined factors associated with multiple indices of dietary adherence in a group of HD patients, including the role of knowledge of food sources of nutrients or fluid as required to be restricted, and knowledge of the medical complications associated with not restricting those nutrients. Durose et al. (2004) previously reported that, if anything, these forms of knowledge were poorer in patients adhering to their restrictions than in those not adhering. Our findings were similar: knowledge of food sources of restricted nutrients did not differ between adherers and non-adherers, and adherers for PO_4_ restriction had significantly less accurate knowledge of medical complications of PO_4_. K adherers also tended to be less accurate in this knowledge, whereas fluid adherers did not differ from non-adherers.

These findings occurred despite there being evidence that knowledge related to a given nutrient was better in those patients having to restrict that nutrient than those not restricted. Thus, health professionals in the HD unit were by and large successfully educating their patients; unfortunately, this knowledge was not associated with adherence. A possible explanation is that non-adherers tend to acquire greater knowledge through repetition of the information from the whole HD team, due to their repeated failure to adhere; nevertheless, this is in line with evidence that information *per se* is a poor predictor of successful health behavior change (Michie et al., 2011). This finding could also reflect greater preoccupation with the dietary restrictions in patients struggling to adhere, as has been reported previously for fluid adherence (Rosenbaum and Ben-Ari Smira, 1986). These are important points to consider when planning new therapeutic interventions for CKD and HD patients; they need to be more than just “better” or “different” ways/formats of information transfer.

This study performed a detailed examination of several relevant psychological variables in this group of HD patients alongside their nutritional data. The relations between adherence and the psychological variables examined here suggest they may reflect other factors which modulate any relationship between information provision and retention and behavior change. For example, the health locus of control results suggest that adherers have a greater belief in the importance of doctors (for PO_4_ and fluid) or “Other people” (e.g., other health professionals; for K and PO_4_) in their renal health outcomes, and less self-reliance (“Internal” health locus of control (HLOC); for K adherers), than non-adherers. Conversely, it was striking that adherers reported significantly less stress in the previous week, irrespective of their restriction requirements. This extends a previous finding that stress was associated with poor K adherence over 4 weeks (Hitchcock et al., 1992) to PO_4_ and fluid adherence over 3 months. In addition, adherers for all restriction groups scored lower on the sensation seeking personality trait than non-adherers, though against our expectations there were no differences for the other traits.

Patients with CKD and specifically those receiving maintenance dialysis experience a complex set of psychological stressors and unsurprisingly therefore clinically relevant manifestations are common. One recent study of HD patients using the Hospital Anxiety and Depression Scale found that many patients had persistent symptoms of depression (39.6%) or anxiety (31.8%) (Ng et al., 2015). In addition clinical depression is associated with low quality of life and increased mortality in HD patients (Zalai et al., 2012). The cause of these psychological problems is multifactorial but the treatment itself can induce acute anxiety whether by merely attending HD therapy or due to changes in the HD treatment such as changes in personnel (Feroze et al., 2012). It is likely that other aspects of care, e.g., nutritional advice/interventions, also impact on patients’ thinking and thus behavior. A recent study examined a small group (*n* = 52) of HD and PD patients in more detail using a battery of psychological tests including the Profile of Mood States, Nottingham Health Profile, Stress Situation Assessment Questionnaire, Social Appreciation Questionnaire, and the State-Trait Anxiety Inventory (Nowak and Laudanski, 2014): end stage renal failure is seen as a loss and threat, and patients reported higher levels of fatigue/inertia and less energy as well as confusion/bewilderment, which may be related to greater sleep disturbance, compared to healthy controls. This negatively affects quality of life as shown in a small study of 39 HD patients using the SF36 (36-item Short Form Quality of Life questionnaire; Perales-Montilla et al., 2012): HD patients had lower health-related quality of life (HRQOL) levels, and depression was the main predictor of HRQOL. In addition, the degree of concern and the use of passive coping strategies for stress (e.g., helplessness–hopelessness and fatalism) were also associated with lower levels of HRQOL. Thus, when HD patients are worried about their disease and its treatment, and find it difficult to cope with, HRQOL falls, whereas self-efficacy is correlated with higher HRQOL. In turn, self-efficacy is linked to adherence to treatment and health-promoting behaviors. This is the background against which our observations relating to psychological variables and dietary/fluid adherence in HD patients should be assessed.

A few previous studies have attempted to understand adherence to diet/fluid and medication in dialysis patients in terms of psychological variables. One study (Chilcot et al., 2010) assessed illness representations (common sense model) using the Revised Illness Perception Questionnaire in 99 HD patients in the UK to examine fluid adherence: adherent patients had significantly higher timeline perceptions, and, after controlling for relevant demographic variables, also higher consequence perceptions. Another study (Garcia-Llana et al., 2013) examined 30 HD patients in terms of their adherence to HD therapy and adherence to PO_4_ lowering medication and anti-hypertensives in relation to psychological variables including the Beck Depression Inventory, State-Trait Anxiety Inventory, Perceived Stress Scale, and the SF36. No differences regarding adherence to antihypertensive or PO_4_ lowering medication and the psychological variables measured were observed. A more recent study provides important findings around perceived (“Internal”) control and adherence to fluid restriction (Howren et al., 2016a). This study examined patients participating in a randomized trial of behavioral self-regulation in 119 HD patients who were not fluid adherent. Perceived control was measured using the Multidimensional Health Locus of Control “Internal” scale and preference for control through the Krantz Health Opinion Survey. The study showed that patients with both high perceived control and high preference for control demonstrated the greatest fluid adherence, and echoes an earlier study reporting that higher levels of perceived self-efficacy and control were associated with fluid adherence (Rosenbaum and Ben-Ari Smira, 1986). In contrast, we found no difference in perceived control (Internal) for fluid adherence, but a greater belief in locus of control in “Doctors.” This was also the case for PO_4_ adherers, whereas K adherers were lower in perceived self-control. These differences might reflect variations in patient samples and settings across studies, but could also reflect different mechanisms acting on adherence to the various dietary requirements.

Planning interventions to support patients with adherence to fluid/diet limitations is challenging, and a review of intervention studies (Welch and Thomas-Hawkins, 2005) found a small number of studies, frequent methodological issues and lack of a robust theoretical model to guide interventions. The recent study (Howren et al., 2016a) of fluid adherence suggests that those patients who believe health-related outcomes are a function of their own behaviors and that they have the opportunity to exert control could be most adherent to complex regimes. But the difficulty remains – how to influence and modulate behavior with interventions in patients with a wide range of beliefs about perceived locus of control.

Although the design of this study does not allows us to infer causality, the observed links between both stress and sensation seeking and dietary adherence might imply psychological predispositions in non-adherers to comfort eating during negative emotions (Gibson, 2012): this would be a worthwhile research question for a subsequent study. Future research could also benefit from using other measures known to influence food choice and intake, such as disinhibited or uncontrolled eating and restrained eating tendencies, as measured by the Dutch Eating Behaviour Questionnaire for example (van Strien et al., 1986), as these attitudes can interact with stress in quite complex ways (Gibson, 2012). However, although early studies of stress and eating showed that stress could increase, or at least not suppress, food intake in restrained eaters as measured by the Restraint Scale (Polivy et al., 1994), the use of more psychometrically consistent restraint scales that do not include emotional items has not replicated those earlier findings for dietary restraint *per se* (Oliver et al., 2000; Lowe and Kral, 2006; Gibson, 2012). Rather, as a measure of strong cognitive control over eating, it is possible that adherers would be higher in restraint than non-adherers. Given that sensation seeking was lower in adherers for all indices, one might expect that adherers would also be lower in uncontrolled eating; yet, impulsivity was not significantly associated with adherence here.

The results of the present study confirm the complexity of behaviors in HD patients which needs to be considered in clinical practice and when designing new therapeutic interventions aiming to improve dietary adherence. Interventions which simply provide information or which are associated with increased stress during delivery are unlikely to be successful. Yet, there is a lack of consistent evidence to support particular intervention strategies to change dietary behavior based on established theoretical models (Prestwich et al., 2014). Desroches et al. (2013) conducted a Cochrane systematic review of varying interventions to improve dietary adherence for a range of chronic diseases and concluded that the evidence was too inconsistent to make firm recommendations. For HD patients, interventions based on education have been shown to improve knowledge but not adherence (Wells, 2011). In this patient group, there are few interventions based on psychological theories related to behavior change, and so far mixed results that fail to support the efficacy of the theory-driven approaches chosen (Molaison and Yadrick, 2003; Wileman et al., 2014). Interventions may need to be tailored for particular patients; moreover, it is clear from our results that the different members of the renal multidisciplinary team all need to play important roles as the locus of control data point to differential impact on adherence among patients.

Limitations of this study include the sample size, being a single center study and the homogeneous ethnicity of the patient sample, which restrict the generalizability of the findings. However, the current study was of similar size to other HD patient studies and the study did examine knowledge of fluid/diet, and understanding of the complications of fluid/diet adherence, alongside actual objective adherence behaviors across K, PO_4_, and fluid criteria, as well as a wide range of psychological variables. This thorough and complete assessment contrasts with many previous studies which have examined only some of these issues. Nevertheless, given the large number of variables examined, a larger scale replication is needed to allow more fine-grained multivariate analyses to confirm our key findings.

In conclusion, knowledge about food sources, and medical complications, of restricted nutrients (K, PO_4_, and fluid) was either not associated with dietary adherence or, in the latter case, was lower in adherers than non-adherers. This may suggest reverse causation, whereby non-adherence leads to more attempts by health professionals to impart relevant knowledge, though it would seem to little avail. In addition, several psychological variables, including beliefs about health locus of control, recent stress, and a sensation seeking personality trait, proved sensitive to dietary adherence status. One conclusion from these psychological associations with dietary adherence in HD patients is that a useful strategy may be to develop a brief screening questionnaire incorporating assessment of such factors, with a view to predicting which patients may be at risk from which form of poor dietary adherence, and therefore may need additional support. Such tailored support could then be targeted more rapidly and specifically in a patient-centric approach to care.

## Author Contributions

EG led the design and data analysis, and was the primary author. IH oversaw ethics approval, patient recruitment and data management, and contributed to drafting the manuscript. DK recruited and tested patients, and contributed to data analysis and drafting the manuscript. PR advised on design, revised the manuscript, and provided important intellectual input during drafting.

## Conflict of Interest Statement

The authors declare that the research was conducted in the absence of any commercial or financial relationships that could be construed as a potential conflict of interest.
