# Erratum: Differences in Knowledge, Stress, Sensation Seeking, and Locus of Control Linked to Dietary Adherence in Hemodialysis Patients

**DOI:** 10.3389/fpsyg.2016.02076

**Published:** 2016-12-27

**Authors:** Frontiers Production Office

**Affiliations:** Frontiers Production Office, FrontiersSwitzerland

**Keywords:** hemodialysis, adherence, diet, stress, locus of control, personality

Reason for Erratum:

Due to a typesetting error, in Table 5, the 2nd column should have been headed “Adherent” instead of “B”.

**Table 5:** **Comparison of personality traits between adherers and non-adherers for potassium, phosphate, and fluid restrictions (mean ± sd)**.

**Restriction**	**Adherent**	***n***	**Sensation seeking (range 6–24)**	**Impulsivity (range 5–20)**	**Anxiety sensitivity (range 5–20)**	**Hopelessness (range 7–28)**
Potassium	Yes	23	11.0^*^ ± 3.5	10.1 ± 3.2	13.8 ± 2.8	15.0 ± 3.2
	No	15	13.5 ± 3.8	11.3 ± 2.8	12.9 ± 1.8	15.3 ± 4.1
Phosphate	Yes	28	10.8^**^ ± 3.1	10.7 ± 2.9	13.8 ± 2.6	14.0 ± 3.1
	No	17	13.3 ± 3.4	11.8 ± 2.5	13.1 ± 2.0	16.0 ± 3.4
Fluid	Yes	14	9.1^**^ ± 2.9	9.4 ± 2.3	13.3 ± 3.0	13.9 ± 3.2
	No	25	12.2 ± 3.9	11.4 ± 3.2	13.6 ± 2.1	14.4 ± 3.4

* *p < 0.05*,

** *p < 0.01 (1-tail, adherers vs. non-adherers). For all personality variables, higher scores indicate greater levels of those traits*.

The publisher apologizes for this error and the correct version of the table appears below. This error does not change the scientific conclusions of the article in any way.

